# A comparative in vitro study of the effect of biospecific integrin recognition processes and substrate nanostructure on stem cell 3D spheroid formation

**DOI:** 10.1007/s10856-020-06373-x

**Published:** 2020-03-23

**Authors:** Valeria Perugini, Matteo Santin

**Affiliations:** Centre for Regenerative Medicine and Devices, School of Pharmacy and Biomolecular Sciences, University of Brighton, Brighton, BN2 4GJ UK

## Abstract

The in vitro study of the properties of the human mesenchymal stem cells as well as their manipulation in culture for clinical purposes depends on the elimination of artefacts caused by the lack of their natural environment. It is now widely accepted that mesenchymal stem cells should be studied when they are organised as 3D spheroids rather than fibroblast-like colonies. Although this can be achieved with the use of some extracellular matrix proteins or by non-adherent conditions these suffer of significant limitations. The recent development of synthetic substrates resembling the physicochemical and biochemical properties of the adult stem cell niche has prompted questions about the role played by nanotopography and receptor-mediated adhesion. In the present paper, the influence of two types of substrates bearing the same nanostructure, but exposing either a non-specific or an integrin-specific binding motif was studied. Carboxybetaine-tethered hyperbranched poly(ɛ-lysine) dendrons showed that the hyperbranched structure was fundamental to induce spheroid formation, but these were forming more slowly, were of reduced size and less stable than those growing on substrates based on the same hyperbranched structures that had been functionalised at their uppermost branching generation by a laminin amino acid sequence, i.e. YIGSR. The study shows that both nanostructure and biorecognition need to be combined to achieve a substrate for stem cell spheroid formation as that observed in vivo in the adult stem cell niche.

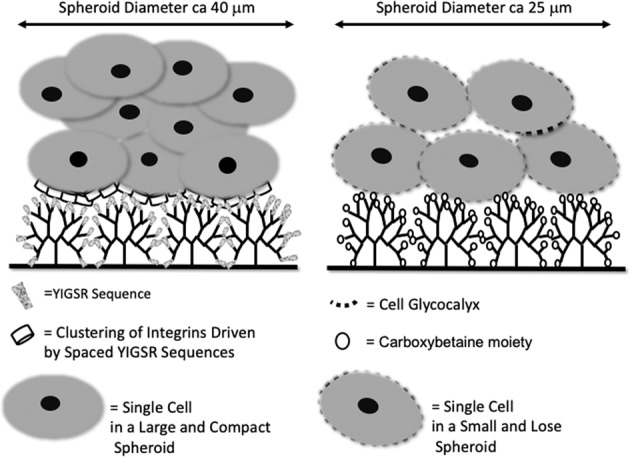

## Introduction

A consensus has been building on the advantage of growing stem cells, such as the human adult bone marrow mesenchymal stem cells (hMSCs), as 3D spheroids rather than as fibroblast-like colonies [1]. This shift in culturing procedures is supported by the tendency of these stem cells to lose their pluripotent phenotype when spreading on tissue culture dish plastic [2]. Conversely, 3D spheroids closely resemble the organisation of this type of cells when nested in their natural in vivo niche expressing higher levels of relevant markers [3].

To date, the induction of 3D spheroidal structures has been pursued either by the use of proteins found in the niche (e.g. laminin) or by non-adherent substrates and microdroplet setting facilitating cell-to-cell interactions rather than cell-substrate adhesion or by substrate topography forcing cell proliferation into the 3D volume rather than on a 2D plane [4, 5].

More recently, the research group at the Centre for Regenerative Medicine and Devices, University of Brighton has developed a synthetic substrate where hyperbranched poly(ɛ-lysine) dendrons expose the amino acid sequence, YIGSR, that is present in laminin (i.e. PhenoDrive, Tissue Click Ltd, UK). This substrate was shown to be able to induce the formation of 3D hMSC spheroids through the combination of the bio-specific features of the laminin (the YIGSR sequence) with those of the mesh-like structure of Collagen Type IV, another key component of adult stem cell niches (the hyperbranched structure of the dendrons). The systematic study highlighted a different clustering of the cell surface integrins induced by the orderly-spaced presentation of a laminin-specific amino acid sequence, the YIGSR, that led to an activation of the intracellular biochemical pathway, Rho-A that is known to inhibit the formation of the cytoskeleton and, as a consequence, of the cell spreading. The same study showed that the initial adhesion of the hMSCs on this type of substrate led to cell spreading and it is the subsequent proliferation that originate round-shaped cells resembling the asymmetric division that hMSCs undergo in their natural niche. Noticeably, the results showed that a reduction of the YIGSR density, and therefore their spacing, led to the more familiar spreading of the cells and their proliferation into fibroblast-like colony [6].

However, what the study did not discern was whether nanotopography has any significant influence on hMSC spheroid formation. It was therefore hypothesised that presenting the cells with a substrate with the same nanotopography, but with a different adhesion motif could shed light on the roles that the physical features and bioligand play in this specific organisation of the hMSCs. The opportunity was sought to tether the PhenoDrive end groups with carboxybetaine (CB) rather than YIGSR. A recent study by Perugini et al. demonstrated that the functionalisation of chitosan with a modified amino acid, the CB improved the adhesion of cells on beads in microgravity culturing conditions [7].

CB has been used in a range of biotechnological applications, above all as cryoprotectant [8, 9]. It has been suggested that this modified amino acid has the capacity to form hydrogen bonds with a range of organic and inorganic acids that are likely to be responsible for the ascertained CB properties in supporting the adhesion and proliferation of several types of cells [10–12].

This paper aims to compares the effect of hyperbranched 2D substrates presenting either the YIGSR sequence or carboxybetaine moiety on hMSC adhesion and to discriminate the contribution of nanotopography and biorecognition processes on the formation, morphological features and cohesiveness of hMSC spheroids.

## Materials and methods

### CB and YIGSR dendron synthesis and coupling to linear poly-L-Lysine (polyK)

PhenoDrive substrates (TissueClick Ltd, UK) were prepared as previously described [6]. Briefly, poly (ɛ-lysine) dendrons of three branching generations (gen3K) with an arginine (R) molecule at their molecular root and 16 linear either laminin peptide-sequence (YIGSR) or carboxybetaine (CB) at their uppermost branching generation were synthesised using a modified microwave (Biotage, UK) based solid phase peptide method. This technique, which is identical to that described by Perugini et al. [6], allowed the assembly of both dendrons on a Tenta Gel NH_2_ resin using a fourfold excess of Fmoc-protected amino acids. Briefly, the resin was initially swollen in dimethylformamide (DMF, Fisher-Scientific, UK) for 15 min, then washed and coupled with a Rink amide linker (0.4 mmoles, Iris Biotech GmbH UK) that was previously dissolved in 3 mL DMF supplemented with 0.45 M O-(1H-benzotriazole-1-yl)-N,N,N′,N′-tetramethyluroniumhexafluorophosphate (HBTU, Sigma-Aldrich, UK) and 33% v/v diisopropylethylamine (DIPEA, Sigma-Aldrich, UK). After 5 min at 50 °C, the reaction was stopped and the Fmoc-protecting groups of the linker were removed by the addition of 20% v/v piperidine in DMF at room temperature for 4 min. The deprotection cycle was repeated twice before an ordinate series of Fmoc-amino acids was added allowing the desired dendrons to be achieved. At the end of the synthesis, each dendron was dried and cleaved from resin using a cocktail mixture of 95% v/v trifluoroacetic acid (TFA, Fisher-Scientific, UK), 2.5% v/v, H_2_O and 2.5% v/v triisopropylsilane (Sigma-Aldrich, UK) at room temperature for three hours. Cleaved dendrons were then precipitated in a large excess of ice-cooled diethyl ether and centrifuged (dend Denley BS400) for three times at 3500 rpm for 5 min.

The identification of both Rgen3K(CB)_16_ and Rgen3K(YIGSR)_16_ was carried out using an electrospray/ionisation time of flight, ESI-TOF MS (Bruker microTOF) at 0.4 bar while their purity was assessed by a standard analytical HPLC (Agilent Infinity).

Afterwards, the purified dendrons were dissolved in 0.1 M MES (Sigma-Aldrich, UK) buffer solution and grafted onto poly-L-lysine hydrobromide (polyK, MW = 70–150 K, Sigma Aldrich, Catalogue number P6282) at a final concentration of 0.01% v/w through the activation of the R carboxyl groups by 4 mM sulfo-NHS and 10 mM EDC (Sigma-Aldrich, UK). After an overnight incubation, the solution was filtered to remove impurities and assessed by Fourier-transform infra-red spectroscopy (FT-IR, Perkin Elmer Spectrum 65) before being used to coat 24 tissue culture wells (Nunc, UK).

### Tissue culture plate coating with biomaterial substrates

Polystyrene tissue culture plates (TCP) were coated with PhenoDrive by a film casting technique. The freeze-dried biomaterial powder was dissolved in ethanol/water (75:25 v/v) solution at a final concentration of 0.01% w/v. Coating was performed by pipetting 150 μL of either non-modified PolyK or PhenoDrive in to each well of 24-well TCP. The solution was left to evaporate at room temperature under sterile laminar flow air conditions overnight. Prior to use, the coated-wells were rinsed thoroughly with distilled water and sterilised by a 256-nm wavelength UV lamp (Perkins) in a sterile tissue culture hood for 1 h.

## A non-modified PK was also prepared as control using the same procedure

### hMSC culture on different substrates

Frozen hMSCs (P2, Lonza Germany) were re-suspended in 5 mL of serum free culture medium (Lonza, Germany) and centrifuged at 500 *g* for 5 min. Cells were then counted, cultured directly onto both tissue culture plate wells (TCP, Nunc, UK) and distinct modified PK substrates at a density of 7000 cells/cm^2^ and maintained at 37 °C in a humidified atmosphere containing 5% CO_2_ for 7 days.

### Spheroid characterisation after culturing on different substrates

During the 7 days of culture, the morphology of hMSCs was periodically assessed under a phase contrast microscope (Leica DM2500) with ×10 objective lens; whereas their stem marker regulation was investigated by staining cells with primary antibodies against Oct4 and Nanog. The cells were fixed and permeabilized in chilled methanol for 10 min and then treated with a solution of 1% w/v bovine serum albumin (BSA, Sigma-Aldrich, UK) to block unspecific sites and immunolabelled with FITC or TRICT-conjugated secondary antibodies (Fisher-Scientific, UK) at a dilution of 1:100 at room temperature for 1 h. Stained hMSCs were finally imaged with a laser scanning confocal microscopy (Leica TCS SP5, UK).

To calculate the average in number of hMSC spheroids and their diameter, phase contrast images were captured at lower magnification (scale bars = 100 µm) and analyzed by NIH ImageJ. The measurements were replicated at least three times and data expressed as mean × standard deviation (*n* = 9).

### Spheroid stability evaluation after biochemical and mechanical detachment

Spheroid detachment was initially performed by tapping or gently agitating the hMSCs-seeding plates for 3, 5 and 20 min. However, spheroids were only harvested after being scrapped and centrifuged at 500 *g* for 5 min. These were then washed twice with phosphate buffer-saline solution (PBS, Aldrich-Sigma, UK), re-plated onto 24-TCP wells and observed using a light microscope (Leica DM2500) after 2 h incubation.

## Results and discussion

**Fig. 1:**
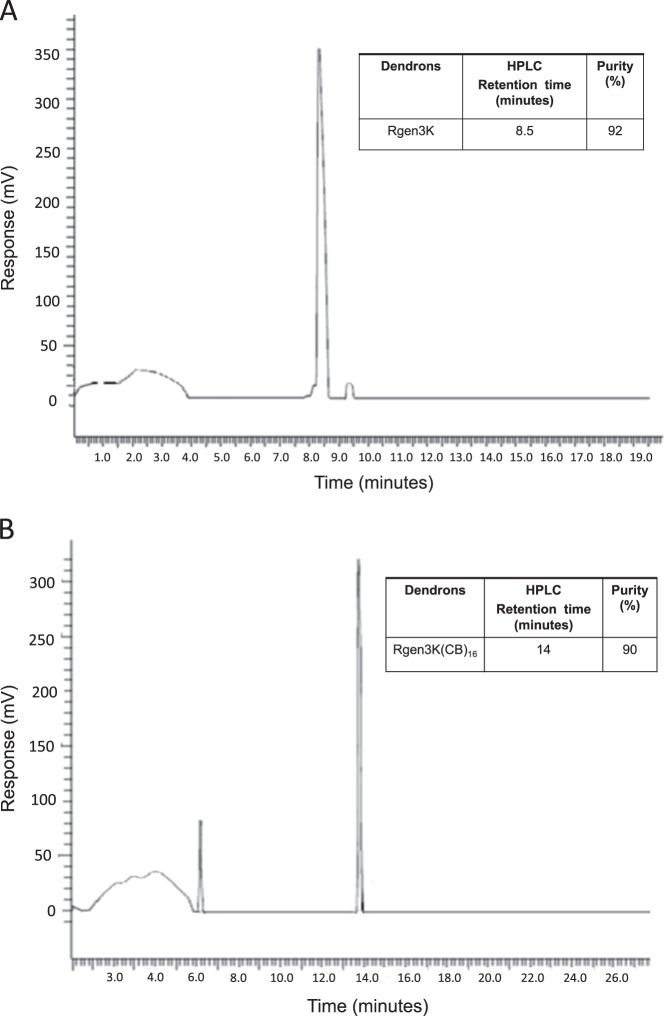
HPLC of non-modified (**a**) and CB-modified (**b**) dendrons

HPLC showed the high degree of purity of the synthesised polyK dendron before and after functionalisation of the amino terminals with CB. These results were comparable to those obtained from the characterisation of the YIGSR-tethered dendrons as previously reported (Fig. 1a, b). When compared to the chromatogram of a non-functionalised dendron, the elution profile of the CB-tethered dendron maintained a high degree of homogeneity, but the elution time was significantly delayed from 8.5 min to 14 min. As the separation medium of the HPLC column was based on a C-14 hydrophobic medium, it can be speculated that the delayed elution of the CB-tethered dendron was due to the known ability of CB to form both inter- and intramolecular hydrogen bonding. This would lead to the formation of larger complexes exposing relatively hydrophobic moieties and masking the interacting hydrophilic functional groups thus leading to stronger adsorption onto the column bead surface.

The purity of the two types of dendrons was 95% for RGen3K and 90% in the case of the RGen3K(CB)_16_ dendrons, respectively. The chromatogram for both types of dendrons showed early broad peaks ascribed to the mobile phase solvents used. A small sharp peak was detected for the RGen3K at a delayed elution time of approximately 10 min suggesting the presence of complexes of dendron rolling along the column. In the case of the RGen3K(CB)_16_ dendron, a very early peak in proximity to the solvent peaks was observed suggesting the presence of free CB molecules not grafted to the dendron. The presence of free CB in the sample was deemed to be irrelevant in the following grafting of the dendron to the linear polyK. This was proven by the significantly different behaviour of the stem cells when cultured on substrates where CB molecules where directly grafted on linear polyK rather than on dendrons (Fig. 3, CB panel, arrow). Here, only rarely and slowly stem cells spheroids were formed.

As expected, the FT-IR characterisation of the non-functionalised and CB-functionalised dendrons showed similar profile, but clear changes in the ratio of some of the peaks in the amino group region, 650–1700 nm^−1^ (Fig. 1a, arrows), with some of the peaks in the CB-functionalised dendrons being attenuated. It is suggested that these changes are caused by the CB masking of the primary amino groups exposed at the N-terminal ends of the branched structure.

To ensure the formation of surface stably coatings on the tissue culture plastic, the two types of dendrons were coupled to the lateral amino groups of a recombinant linear poly-L-Lysine (PolyK).

**Fig. 2:**
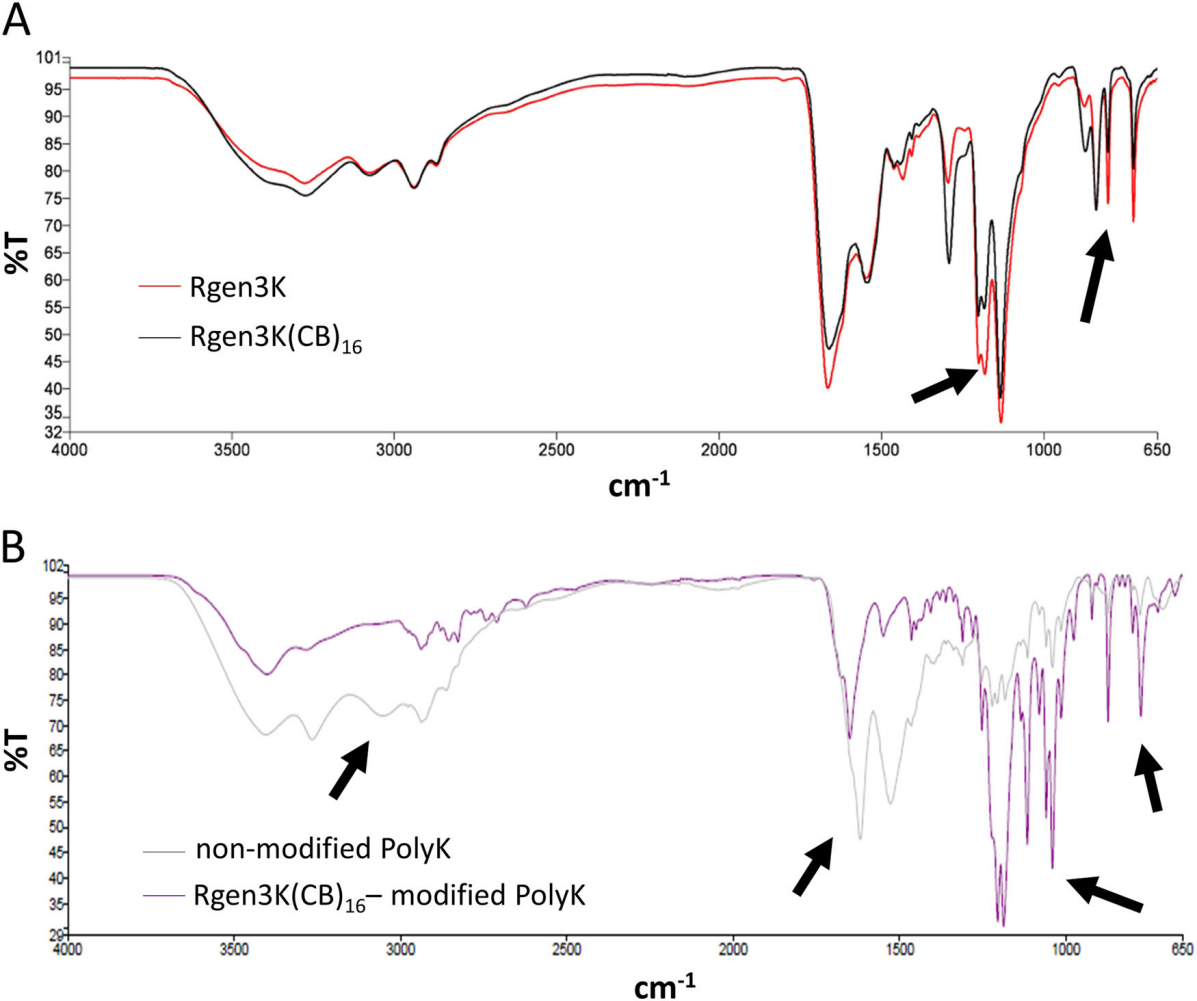
FTIR of non-modified and CB-modified dendrons (**a**) and their grafting on linear polyK (**b**)

FT-IR analysis showed even clearer differences in the typical amino group regions, 650–1700 nm^−1^ and 2500–3500 nm^−1^ when the spectrum of a linear polyK was compared to the same biopolymer after its modification with CB-tethered dendrons (Fig. 2b, arrows). Most of these changes were attributed to the increased presence of both primary and secondary amine introduced by the coupling of dendrons on the relatively simpler structure of the linear polyK.

The size of the non-modified polyK was not expected to induce any difference in the cell behaviour that was previously shown to be determined by the effect of the dendrons on the cells [6]. A linear polymer with a reported MW 70–150 K was chosen to ensure a stable adsorption of the coating on the TCP polystyrene surface.

**Fig. 3:**
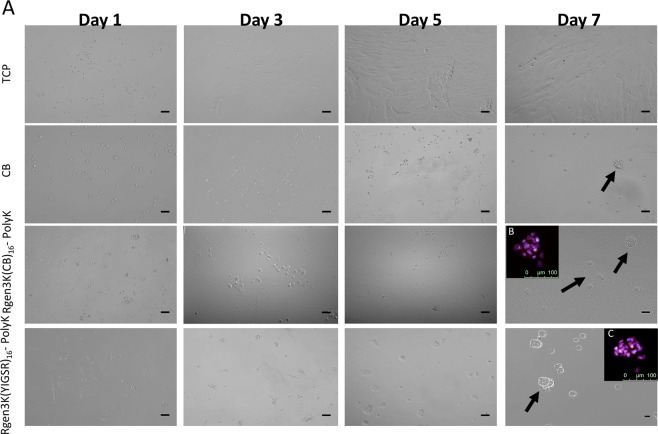
Light microscopy of hMSC proliferation on different substrates over 7 days of culture. Arrows indicate spheroid formation. Inserts B and C show OCT-4 (green staining) and NANOG (red staining) immunocytochemistry (merged images = purple staining). (Scale bar is 100 µm)

hMSCs were cultured in serum-free medium over 7 days on TCP, CB-modified linear polyK, CB-tethered dendrons coupled on linear polyK and on the same linear polyK when modified with YIGSR-tethered dendrons (Fig. 3). Light microscopy showed that cells grew as single spread cells on TCP forming the well-known fibroblast-like colony units. When CB was used to modify the linear polyK, the cells acquired a round-shaped morphology and sporadically formed spheroids (Fig. 3, panel second row, arrows). These results clearly demonstrated the different effect generated by the presence of CB in the substrates on cell behaviour. In particular, this part of the study confirmed that CB encourages cell adhesion, but that it prevents spreading [13, 14].

**Fig. 4:**
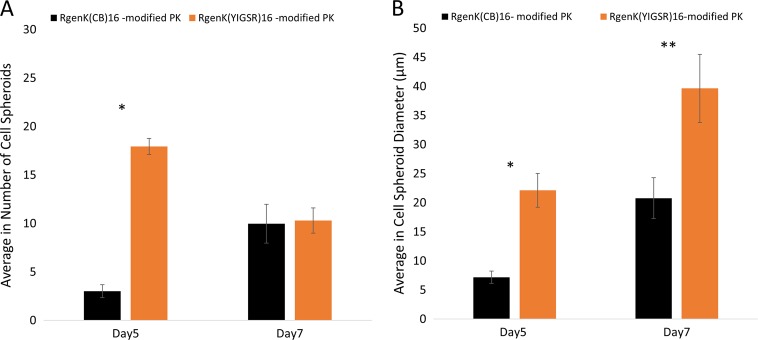
Characterisation of hMSC spheroid numbers (**a**) and size (**b**) at 5 and 7 days of culture. (*P ≤ 0.01; **P ≤ 0.001; mean ± SD; *n* = 3)

The comparison with substrates presenting a hyperbranched structure clearly encouraged the formation of 3D spheroids, more significantly after 7 days of culture (Fig. 3, panel third and forth row, arrows). These spheroids expressed typical transcriptional factors present in progenitor cells, OCT-4 and NANOG (Fig. 3, panel inserts A and B). In the merge images, the red and green fluorophore tagging of the respective antibodies for these two markers overlapped appearing as purple staining. The quantification of the size and number of spheroids was quantified as a measure of their proliferation rate clearly showing that the biorecognition processes offered by the exposure of the YIGSR bioligand to the cell integrin led to significantly higher numbers and larger spheroids both at 5 and 7 days (Fig. 4a, b). These results supported the choice of the laminin-mimicking YIGSR sequence as specific bioligand to mimic the basement membrane of the stem cell niche. Indeed, laminin is known to be one of the main components of the basement membrane and to induce the formation of stem cell spheroids in vitro that are similar to those found in this work.

Both morphological and quantitative data demonstrate that the branched structure is necessary to induce the formation of spheroids. A previous work by Perugini et al. on YIGSR-tethered dendron substrates showed that reducing the branching generation from 3 generations (i.e. 16 branches per dendron) to 2 generations (i.e. 8 branches per dendron) brought the hMSCs back to spreading and growing into fibroblast-like colonies [6]. In the present paper, the comparison of the same cells when adhering and proliferating on a linear polyK functionalised with CB only or with CB-tethered dendrons clearly showed that spheroids could significantly form only when supported by a hyperbranched structure. Whether this is caused by the high density of the CB moieties or by a rougher nanotopography as previously demonstrated by SEM is not known. However, the comparative study between surfaces of the same roughness but exposing non-specific adherent groups (i.e. the CB) or bioligands (i.e. the YIGSR sequence) clearly demonstrated that the biospecific recognition by integrin receptors on the hMSC membrane was significantly favouring the spheroid formation; their proliferation was faster and their size bigger in the case of YIGSR-tethered dendron substrates.

To prove that the formation of the hMSC spheroids was not due to anti-adherent properties, both types of cell cultures underwent steps of gradual mechanical solicitation by tapping and orbital shaking for 3, 5 and 20 min. None of these treatments led to the detachment of the spheroids on both CB-functionalised and YIGSR-functionalised polyK substrates (data not shown).

**Fig. 5:**
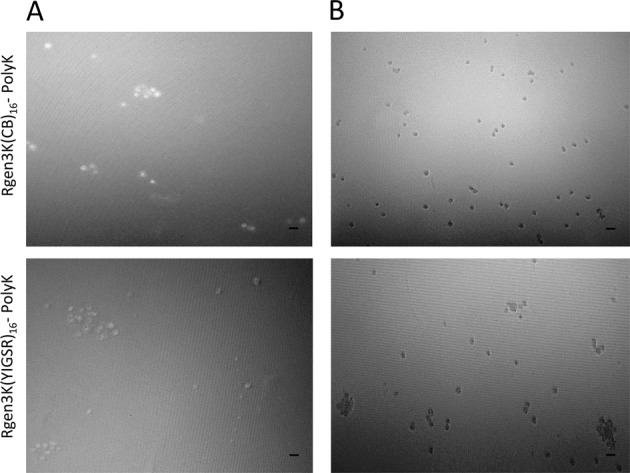
Light microscopy of spheroids detached by scraping from different substrates and re-seeded on tissue culture plate immediately and 2 h after seeding. (Scale bar is 100 µm)

The proof of stable adhesion was followed by a test of spheroid cohesiveness by biochemical (i.e. conventional trypsinisation step) and mechanical detachment (i.e. scraping). After 10 days in culture, the trypsinisation of the spheroids adhering on both substrates led to their dissociation into single cells (data not shown). When subject to scraping and re-seeding on uncoated tissue culture plates in serum-enriched medium (Fig. 5a, b), the very early observation (i.e. immediately after re-seeding and after 2 h incubation), clearly showed that hMSCs previously grown as spheroids on CB-tethered dendron substrates were deposited as lose spheroids, but later completely dissociated adhering on TCP as single round-shaped cells (Fig. 5, panel top row, a and b). In the case of the spheroids removed by scraping from the YIGSR-tethered dendron substrates, spheroids appeared less cohesive, but still clearly visible throughout the plate after 2 h despite contacting the plastic substrate that is known to facilitate hMSC spreading (Fig. 5, panel bottom row, a and b). These experiments suggested that after 10 days in culture spheroids formed on YIGSR-tethered dendron substrates may have started the synthesis and deposition of extracellular matrix holding them together more strongly. Conversely, spheroids formed on CB-tethered dendron substrates appear to have been less active in depositing their own extracellular matrix and therefore unable to compete with the predominant forces of serum proteins adsorbed on TCP surface in conventional tissue culture conditions [15]. The data of this work contribute to define the fundamental characteristics both of the adult stem cell phenotype and of the micro-environment in which it can be preserved [16, 17]. They also contribute to study stem cells in vitro through the development of models that can ascertain their contribution to healthy and disease conditions [18].

It is known that, in their natural niche, stem cells divide asymmetrically leading to the formation of 3D clusters and ensuring, at the same time a reservoir of undifferentiated cells and a progeny of committed cells [19]. This has prompted a worldwide effort to develop in vitro models favouring the formation of 3D spheroids either forcing the cells towards cell-to-cell interactions in non-adherent plates or through the development of biomaterial substrates [5, 20–24] demonstrating that in such conditions the stem cells can uniquely maintain features otherwise lost during their culturing [4, 25, 26]. However, by inducing cell-to-cell interactions non-adherent plates undermine the important role played by the basement membrane of the natural stem cell niche where the important role of proteins such as the laminin in controlling integrin-mediated adhesion and gene expression has clearly been demonstrated [27–29]. Uniquely, the different formulation of the substrates used in this work enable to evaluate the role of the integrins against that played by a substrate with the same nanotopography but different adhesion motifs thus offering a clear advantage over previous work where comparisons have been made with non-adherent substrates and mechanical drive [5, 21]. Instead, the present work weighs the contribution of biospecific interactions between ligands of the extracellular matrix proteins and cell receptors against that of nanotopography play showing that, while both are important in inducing the 3D spheroid formation, the stabilisation of these structures is mainly a consequence of the receptor-mediated binding that can determine their behaviour including gene expression and intracellular pathway activation [6] as well as proliferation and migration [30].

## Conclusions

The recent discovery that hMSC 3D spheroids can be obtained [31] by their culturing on synthetic substrates presenting biospecific laminin ligands at a high density and nanospaced manner provides a significant technological advantage in the study and manipulation of these cells for research and therapeutic purposes as well as for the in vitro testing of drugs or nanomedicines [32–36]. This paper, shows that, while nanotopography and/or density of relevant moieties are key to the formation of spheroids, the biospecific recognition through bioligands typical of extracellular matrix proteins is fundamental to the reconstruction of a truly biomimetic micro-environment similar to that of the natural adult stem cell niche. The results of this paper show the potential of PhenoDrive as a reliable standard substrate for the controlled culture of stem cells for both cell biology research and cell therapy. It is widely accepted that the fully understanding of the stem cell biology and their future use in cell therapy will depend on the availability of substrates that can ensure their handling in vitro in a way that can preserve their pluripotent phenotype as expressed in the natural niche.
